# Src-mediated morphology transition of lung cancer cells in three-dimensional organotypic culture

**DOI:** 10.1186/1475-2867-13-16

**Published:** 2013-02-14

**Authors:** Hong T Nguyen, Yan Zhuang, Lichun Sun, Steven P Kantrow, Jay K Kolls, Zongbing You, Ying Zhuo, Bin Shan

**Affiliations:** 1 Department of Medicine, Tulane University School of Medicine, 1430 Tulane Avenue, New Orleans, LA 70112, USA; 2 Department of Medicine, Louisiana State University Health Sciences Center, 1901 Perdido Street, New Orleans, LA 70112, USA; 3 Department of Pediatrics, University of Pittsburgh School of Medicine, Pittsburgh, PA 15224, USA; 4 Department of Structural and Cellular Biology, Tulane University School of Medicine, 1430 Tulane Avenue, New Orleans, LA 70112, USA

**Keywords:** TGF-β1, Src, Type I collagen, Three-dimensional culture, Extracellular matrix

## Abstract

A fribotic tumor microenvironment promotes progression of cancer. In this study, we utilize a reconstituted basement membrane mimics Matrigel based three-dimensional organotypic culture (rBM 3-D) to investigate the mechanisms that mediate the tumor promoting effects of the fibrogenic mediators TGF-β1 and type I collagen (Col-1) on lung adenocarcinoma cells. Similar to normal alveolar epithelial cells, the well-differentiated lung adenocarcinoma cells in rBM 3-D culture undergo acinar morphogeneis that features polarized epithelial cell spheres with a single central lumen. Either TGF-β1 or Col-1 modestly distorts acinar morphogenesis. On the other hand, TGF-β1 and Col-1 synergistically induce a transition from acinar morphology into stellate morphology that is characteristic of invasive and metastatic cancer cells. Inhibition of the Src kinase activity abrogates induction of stellate morphology, activation of Akt and mTOR, and the expression of tumor promoting genes by TGF-β1 and Col-1. To a similar extent, pharmacological inhibition of mTOR abrogates the cellular responses to TGF-β1 and Col-1. In summary, we demonstrate that TGF-β1 and Col-1 promote stellate morphogenesis of lung cancer cells. Our findings further suggest that the Src-Akt-mTOR axis mediates stellate morphogenesis. These findings also indicate that rBM 3-D culture can serve as an ideal platform for swift and cost-effective screening of therapeutic candidates at the interface of the tumor and its microenvironment.

## Background

A stiff and fibrotic microenvironment promotes tumor progression in experimental models [1,2]. Accordingly, a fibrotic stroma is an independent prognostic indicator of metastasis and poor prognosis [3]. The majority of such evidence comes from the investigation of breast cancer in which the aberrantly stiff extracellular matrix (ECM) is a well-established risk factor [4]. A recent study has provided mechanistic insight into the link between the stiff ECM and progression of breast cancer [5]. Lysyl-oxidase (LOX) increases the stiffness of ECM via crosslinking collagen and thereby enhances integrin signaling to promote invasion and metastasis [5]. Recent advances in lung cancer research implicate a similar presence and function of a fibrotic tumor microenvironment. The expression of transforming growth factor-β1 (TGF-β1) and type I collagen (Col-1), two of the most potent fibrogenic mediators in the lung, is up-regulated in human lung cancer and overexpression of the two can promote invasion and metastasis in experimental models of lung cancer [6-8]. Elevated expression of LOX is a biomarker of invasion and an independent predictor of poor prognosis in patients with early stage lung adenocarcinoma [9]. In experimental models of lung cancer, LOX promotes tumor progression and is targeted by the tumor suppressor gene LKB1 [10]. However, the molecular mechanisms that mediate tumor progression promoted by the fibrotic tumor microenvironment in the lung remain poorly understood.

A substantial amount of our understanding of the tumor modulating functions of the tumor microenvironment has been obtained using three dimensional organotypic culture based on Matrigel, a reconstituted basement membrane mimics (rBM 3-D) [11-13]. rBM 3-D culture faithfully recapitulates salient *in vivo* properties of the epithelium from various tissues. The gene expression signature from rBM 3-D culture of breast cancer cells holds prognostic value for breast cancer [14]. rBM 3-D culture is also a valuable tool to discriminate cancer cells with distinct tumorigenic potential [15]. In general, the non-invasive/metastatic breast cancer cells exhibit a mixture of acinar and mass morphology that features spheroid colonies (mass) with occasional formation of a single central lumen (acinus), whereas the invasive/metastatic cancer cells exhibit stellate morphology that features prominent invasive projections that often bridge multiple cell colonies. More importantly, rBM 3-D culture provides an ideal system to reconstitute the tumor microenvironment for mechanistic investigations. For instance, investigation of Col-1 and its cognate integrin receptors in rBM 3-D culture of mammary epithelial cells has identified the stiff ECM-integrin axis as a driving force of initiation and progression of breast cancer [1,2,5]. Two recent applications of rBM 3-D culture demonstrate its promise in elucidating molecular and cell biology of lung epithelial cells. In rBM 3-D culture, primary human lung alveolar type II cells form alveolar acini [16]. Similar to mammary epithelial cells, alveolar acini exhibit salient differentiation features, such as a polarized monolayer of alveolar type II cells and secretion of surfactant proteins into the central lumen. Because lung adenocarcinoma generally originates from alveolar type II cells, it is plausible that dysregulation of alveolar acini is a pivotal dedifferentiating step in lung tumorigenesis. In support of this concept, over-expression of the tumor suppressive PPAR-γ gene can restore alveolar acini in rBM 3-D organotypic culture of H2122 cells, an aggressive and poorly differentiated human lung adenocarcinoma cell line [17].

Recent advances have shown that the tumor associated stroma and microenvironment are active modulators of tumorigenesis rather than passive bystanders [18]. The current study utilizes rBM 3-D organotypic culture to investigate a link between the behavior of lung cancer cells and the fribrogenic mediators derived from the tumor microenvironment.

## Results

### Morphogenesis of lung cancer cells in rBM 3-D culture

**Figure 1:**
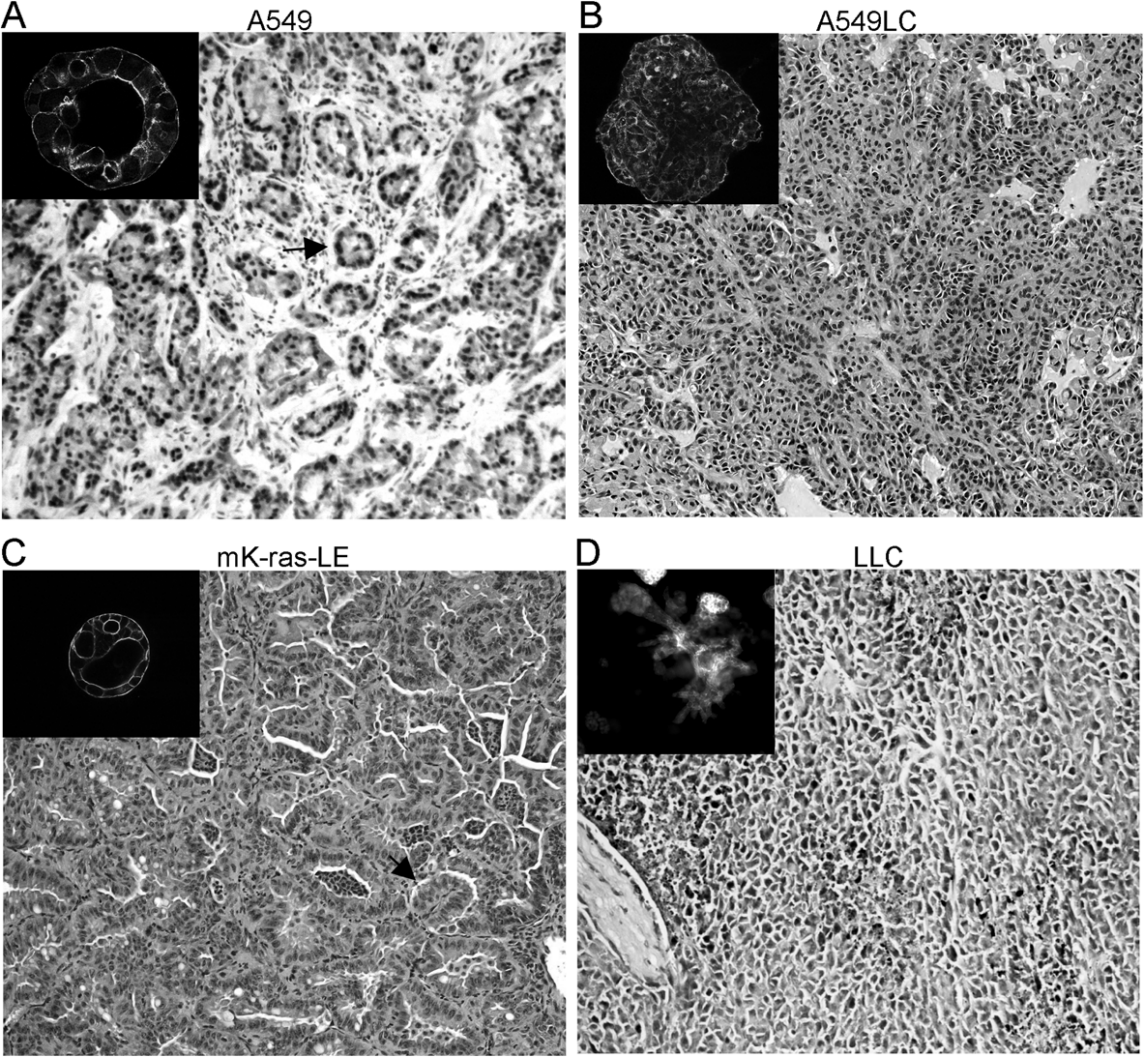
**Distinct growth patterns of lung cancer cell lines in rBM 3-D organotypic culture.** Four human and mouse lung cancer cell lines were cultured in rBM 3-D culture. Morphogenesis of lung cancer cells was visualized by filamentous actin staining and confocal fluorescent microscopy (insets). The insets of **A-C** were captured at 400x and part D was captured at 200x because of the larger diameter of LLC cell cluster. The growth patterns of lung cancer cells *in vivo* were visualized using H & E staining (main images, 100x). Representative glandular structures are indicated by arrows in **A & C**.

rBM 3-D organotypic culture can promote differentiation of lung epithelial cells *in vitro*[16,17]. Therefore, we utilized this model to examine the effects of the fibrogenic mediators from the tumor microenvironment on morphogenesis of lung cancer cells. We established rBM 3-D culture of 4 human and mouse lung cancer cell lines with distinct tumorigenic properties. A549 cells are a well-differentiated non-metastatic human lung adenocarcinoma cell line with residual characteristics of alveolar type II epithelial cells [19]. Similar to normal alveolar type II epithelial cells, A549 cells formed acini, a polarized cell sphere with a single central lumen in rBM 3-D culture (Figure 1A, inset) [16]. Moreover, acini formed by A549 cells in rBM 3-D culture resembled the glandular histology observed in the tumors formed by the implanted A549 cells in mice (Figure 1A). In contrast, A549LC cells, a more aggressive derivative of A549 cells (see Methods), exhibited mass morphology that featured irregular cell clusters void of a central lumen, which resembled the poorly differentiated H2122 cells in rBM 3-D culture as reported in a previous study (Figure 1B, inset) [17]. In congruence, the A549LC xenografts displayed disorganized structure and lacked the glandular histology (Figure 1B). Moreover, A549LC cells acquired greater tumorigenic activity than A549 cells *in vivo* because the implanted A549LC cells doubled the growth of the implanted parental A549 cells, 0.21 ± 0.04 g versus 0.1 ± 0.03 g with marginal significance (*P* value = 0.0678, n = 7, Additional file 1: Figure S1). We further compared morphogenesis of two murine lung cancer cell lines mK-ras-LE and LLC. mK-ras-LE cells were established from a tumor bearing lung of a K-ras^*LA1*^ mouse, a transgenic strain that develops lung adenocarcinoma with limited metastasis [20]. Consistent with their well-differentiated phenotype, mK-ras-LE cells formed acini in rBM 3-D culture, which correlated with the glandular histology in the tumor formed by the implanted mK-ras-LE cells (Figure 1C). In contrast, the metastatic LLC cells exhibited stellate morphology that is characteristic of metastatic cancer cells (Figure 1D, inset) [15]. The stellate morphology featured irregular cell clusters with extensive intersecting cell protrusions (Figure 1D, inset). In accordance, the implanted LLC cells grew into irregular cell masses at the primary site and metastasized to the lung (Figure 1D and unpublished observations). The correlation of morphogenesis of four lung cancer cell lines in rBM 3-D culture and histology *in vivo* indicated that rBM 3-D culture is an appropriate *in vitro* model to assess morphogenesis that is relevant to tumorigenic behaviors of lung cancer cells *in vivo*.

### Src-mediated stellate morphogenesis induced by TGF-β1 and Col-1

**Figure 2:**
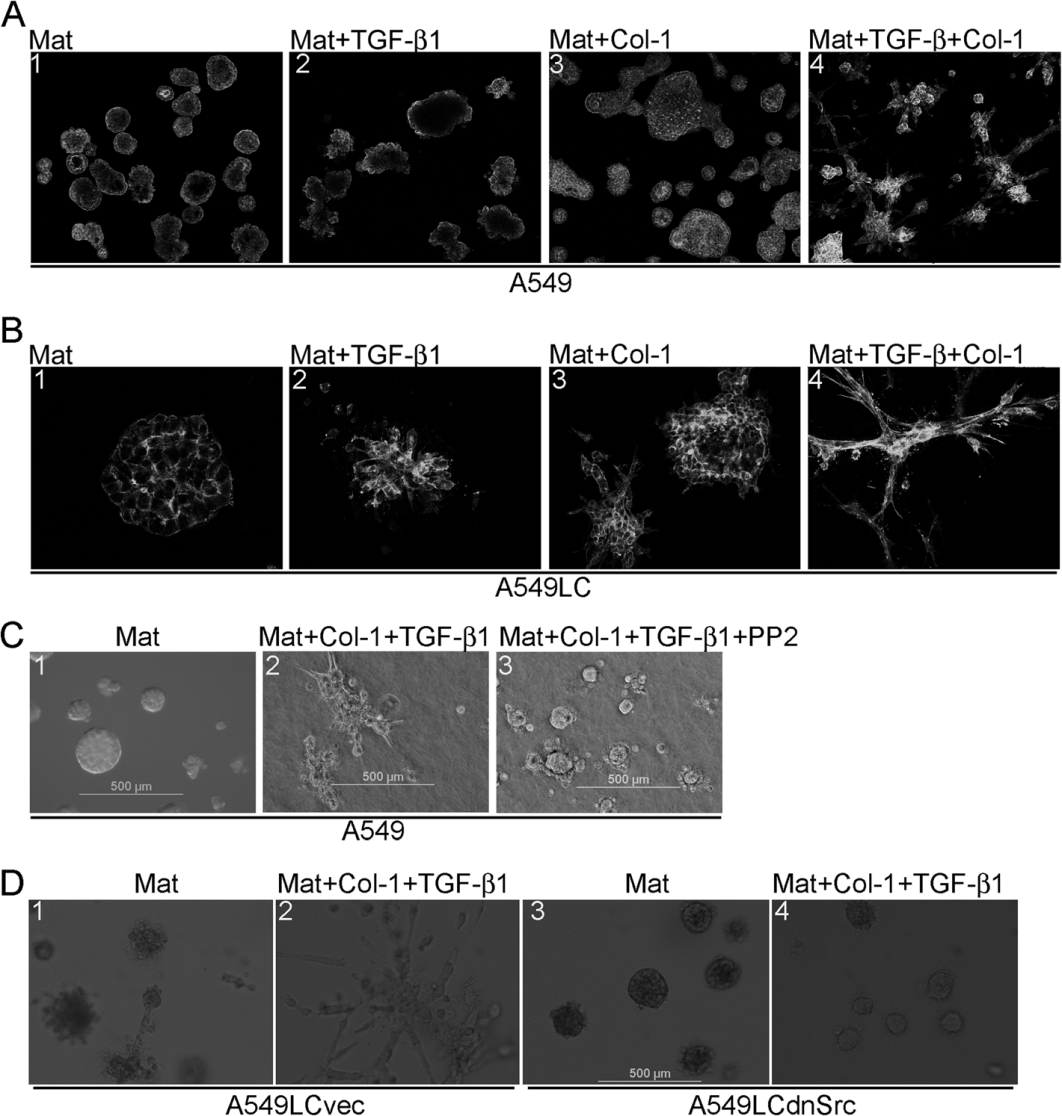
**Induction of stellate morphology by TGF-β1 and Col-1.** A549 and its more aggressive derivative A549LC cells were cultured in rBM 3-D culture (Mat) with or without supplementation of TGF-β1 and/or Col-1. **A)** Morphogenesis of A549 cells in various culture conditions were visualized by filamentous actin staining and confocal fluorescent microscopy. The images were captured at 100x. **B)** Similar to part A except that the images were captured from A549LC cells at 200x. **C)** Phase-contrast microscopy was used to capture images of A549 cells cultured in rBM 3-D (Mat) exposed to the indicated combinations of TGF-β1, Col-1, and PP2 (100x). **D)** The variants of A549LC cells, A549LCvec and A549LCdnSrc were cultured in rBM 3-D (Mat) in the present or absence of TGF-β1+Col-1 and morphogenesis was monitored as described in part C.

Because the tumor microenvironment is commonly fibrotic and enriched with fibrogenic mediators, we examined morphogenesis of A549 and A549LC cells in rBM 3-D culture exposed to various combinations of TGF-β1 and Col-1, two prominent fibrogenic mediators in the tumor microenvironment [6-8]. Addition of TGF-β1 (5 ng/ml) or Col-1 (1.5 μg/ml) alone caused little to modest perturbation of acini as evidenced by distorted cell clusters and partial to complete filling of central lumens (Figure 2A). Simultaneous exposure to TGF-β1 and Col-1 abolished acinar morphology and induced a transition into stellate morphology that was characteristic of invasive/metastatic cancer cells (Figures 2A &1D) [15]. In a similar fashion, A549LC cells underwent transition from mass morphology into stellate morphology upon simultaneous exposure to TGF-β1 and Col-1 in rBM 3-D culture (Figure 2B).

The Src kinase is a key signal transducer of ECM and growth factors [21]. We then questioned whether the Src kinase activity is required for induction of stellate morphology by TGF-β1 and Col-1. To this end, A549 cells were exposed to TGF-β1 and Col-1 in the presence or absence of PP2 (5 μM), an Src selective inhibitor. When compared to the group treated with the DMSO vehicle, PP2 abrogated induction of stellate morphology by TGF-β1 and Col-1, but did not restore acinar morphology because the cell colonies were still void of a single central lumen (Figure 2C). Similar observations were made in A549LC cells upon exposure to various combinations of TGF-β1, Col-1, and PP2 (data not shown). To further confirm a requirement of the Src kinase activity for induction of stellate morphology by TGF-β1 and Col-1, we generated two variants of A549LC cells that were transduced with either a retroviral vector expressing a dominant-negative Src mutant (A549LCdnSrc) or its backbone vector (A549LCvec). Similar to PP2, the expression of the dnSrc mutant abolished stellate morphology induced by TGF-β1 and Col-1, whereas A549LCvec’s response to TGF-β1 and Col-1 was comparable to that of the parental A549LC cells (Figure 2, B & D). These findings indicated a requirement of the Src kinase activity for induction of stellate morphology by TGF-β1 and Col-1.

**Figure 3:**
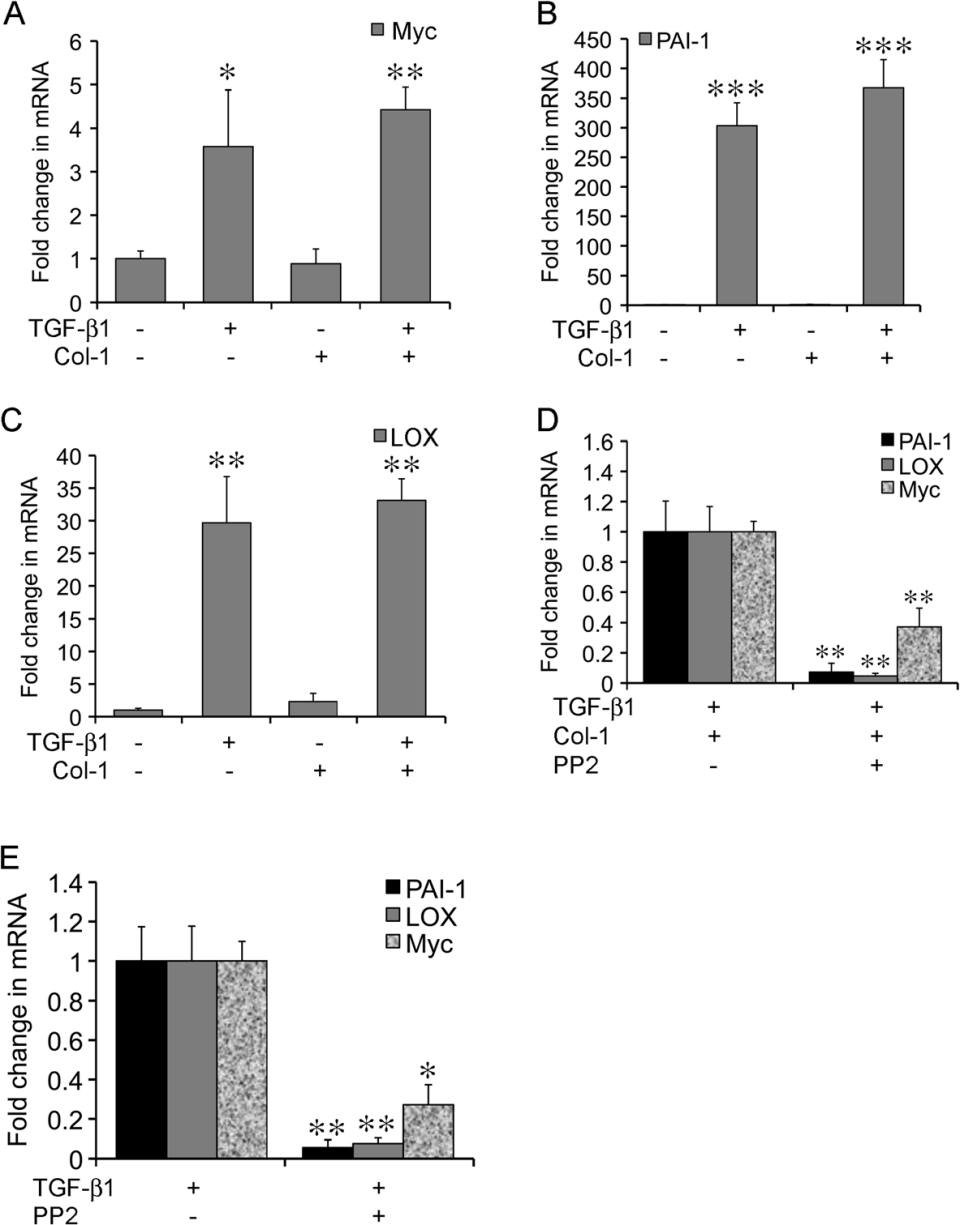
**Activated expression of the tumor promoting genes by TGF-β1 in rBM 3-D.** Total cell RNA was extracted from A549 cells cultured in rBM 3-D (Mat) culture exposed to the indicated combinations of TGF-β1 and Col-1. The expression of the selected tumor-promoting genes was determined using quantitative RT-PCR. Ratios of each mRNA over the housekeeping gene 36B4 were compared across various culture conditions. **A-C)** The expression of Myc, PAI-1, and LOX was evaluated, respectively. A fold change was obtained by setting the values from the control group to one. **D)** The expression of Myc, PAI-1, and LOX was compared between A549 cells cultured in Mat+TGF-β1+Col-1 with or without PP2. A fold change was obtained by setting the values from the PP2 minus group to one. **E)** Similar to part C except that the expression of Myc, PAI-1, and LOX was compared between A549 cells cultured in Mat+TGF-β1 with or without PP2. Mean and standard deviations were obtained from three independent experiments. *, **, and *** indicate a *P* value < 0.05, 0.01, and 0.001, respectively.

To elucidate the mechanisms underlying induction of stellate morphology, we examined the expression of three tumor-promoting genes, namely Myc, LOX, and plasminogen activator inhibitor-1 (PAI-1) because of their established link to TGF-β1 and Col-1 [5,6,8]. The mRNA levels of these genes were determined using quantitative RT-PCR in A549 cells under various culture conditions. TGF-β1 alone induced a robust increase in the expression of all three genes over the control group (3.6-fold in Myc, 303-fold in PAI-1, and 29.7-fold in LOX) (Figure 3, A-C). In contrast, Col-1 alone did not cause noticeable alteration in the expression of these genes. Despite the synergistic induction of stellate morphology, combination of TGF-β1 and Col-1 did not result in synergistic increase in the expression of these genes (Figure 3, A-C). These findings indicated that activation of the Myc, PAI-1, and LOX genes were by and large driven by the TGF-β1 pathway during transition towards stellate morphology. Because inhibition of Src abolished stellate morphology induced by TGF-β1 and Col-1, we examined the effects of PP2 on the induction of Myc, PAI-1, and LOX by TGF-β1 and Col-1 in rBM 3-D culture of A549LC cells. As expected, PP2 substantially reduced the induction of Myc (reduced to 37%), PAI-1 (reduced to 7%), and LOX (reduced to 5%) (Figure 3D). PP2 also inhibited TGF-β1-induced expression of Myc (reduced to 24%), PAI-1 (redcued to 6%), and LOX (reduced to 8%) (Figure 3E). Similar observations were made in A549LCvec and A549LCdnSrc cells (data not shown). These findings indicated a requirement of the Src kinase activity for induction of the Myc, PAI-1, and LOX genes by TGF-β1 in rBM 3-D culture.

### Activation of the Akt-mTOR axis

**Figure 4:**
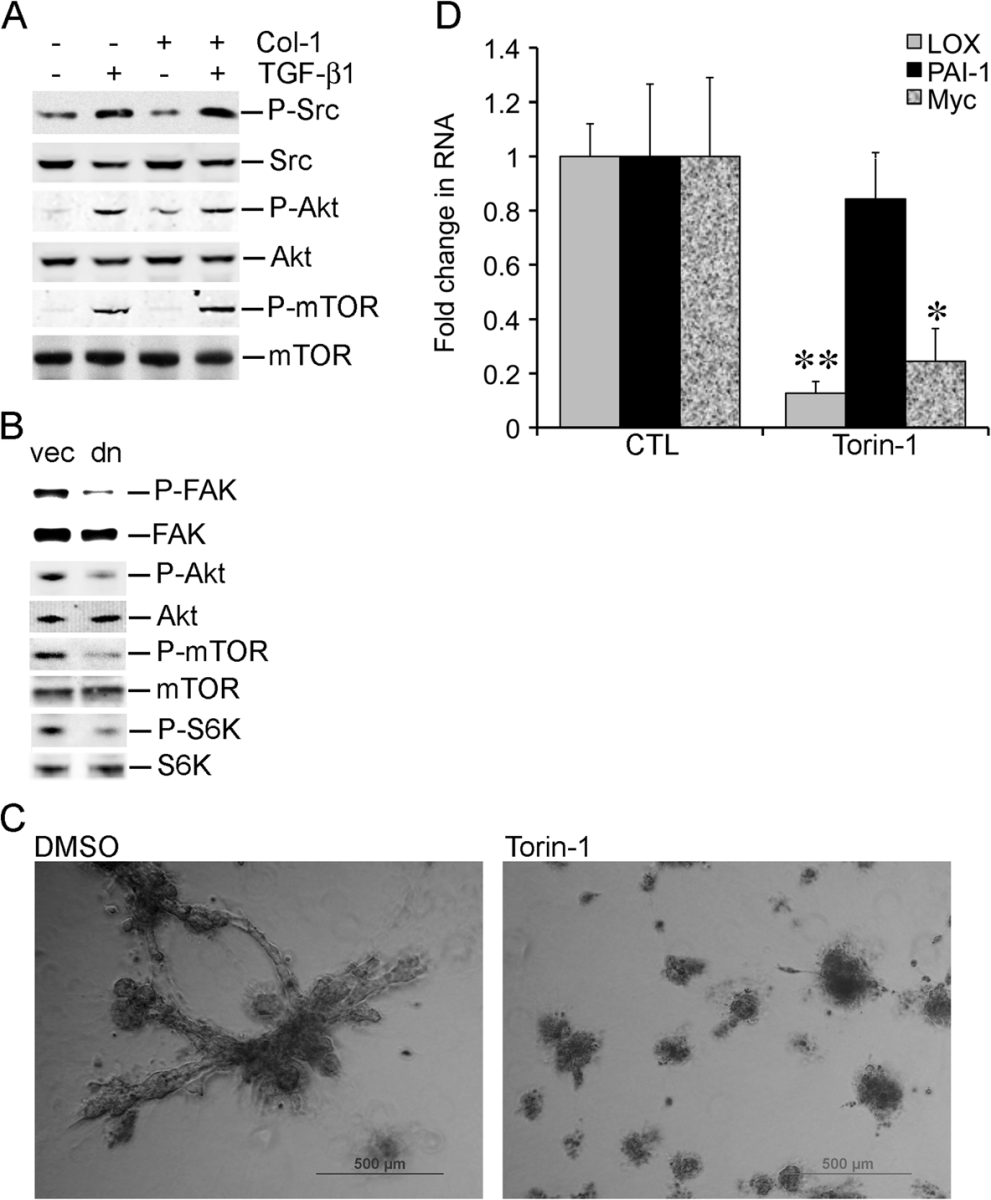
**Activation of the Akt-mTOR axis in stellate morphogenesis. A)** Total cell protein was extracted from A549 cells in rBM 3-D culture exposed to the indicated combinations of TGF-β1 and Col-1. The protein levels of total and phosphorylated Src, Akt, and mTOR were determined using immunoblots. **B)** Similar to A except that total cell protein was extracted from A549LCvec and A549LCdnSrc cells in rBM 3-D culture exposed to TGF-β1 and Col-1. The protein levels of total and phosphorylated FAK, Akt, mTOR, and p70 S6K were determined using immunoblots. **C)** A549LC cells were cultured in rBM 3D culture supplemented with TGF-β1 and Col-1 in the presence or absence of Torin-1 (250 nM). Morphogenesis of A549LC cells was monitored for 12 days. Images were captured using phase contrast microscopy. **D)** The culture condition was identical to that described in part B. Total cell RNA was extracted from A549 cells. The expression of LOX, PAI-1, and Myc was determined using quantitative RT-PCR. Ratios of each gene transcript over the housekeeping gene 36B4 were compared across various culture conditions. A fold change was obtained by setting the values from the control group to one. Mean and standard deviations were obtained from three independent experiments. * and ** indicate a P value < 0.05 and 0.01, respectively.

Src mediates activation of the Akt-mTOR axis in certain experimental conditions [22,23]. Because the Src kinase activity is required for stellate morphogenesis induced by TGF-β1 and Col-1, we questioned whether the Akt-mTOR axis was activated by TGF-β1 and Col-1 in an Src-dependent manner. TGF-β1 alone activated Src in rBM 3-D culture because TGF-β1 increased phosphorylation of Src at ser416 (Figure 4A). In contrast, Col-1 did not activate Src in rBM 3-D culture (Figure 4A). Moreover, a combination of TGF-β1 and Col-1 did not further increase the levels of Src phosphorylated at Ser416 (Figure 4A). In a similar fashion, the Akt-mTOR axis was activated by TGF-β1 regardless of the presence of Col-1 because Akt became hyper-phosphorylated at Ser473 and mTOR became hyper-phosphorylated at Ser2448, a target site of Akt (Figure 4A). To determine whether the Src kinase activity was required for activation of the Akt-mTOR axis, we compared phosphorylation of Akt and mTOR in A549LCvec and A549LCdnSrc in rBM 3-D culture exposed to TGF-β1 and Col-1 (Figure 4B). The dominant-negative activity of the dnSrc mutant was confirmed as A549LCdnSrc exhibited a reduced phosphorylation at Tyr861 in fo-cal adhesion kinase (FAK), a classical target of Src (Figure 4B) [24]. As expected, A549LCdnSrc cells exhibited a substantial decrease in phosphorylation of Ser473 in Akt (Figure 4B). Consistent with reduced activation of Akt, A549LCdnSrc exhibited reduced phosphorylation of Ser2448 in mTOR (Figure 4B). Lastly, we examined phosphorylation of Thr389 in p70 S6K, a target site of mTOR. The expression of the dnSrc mutant substantially reduced phosphorylation of Thr389 in p70 S6K. These findings indicated a requirement of the Src kinase activity for activation of the Akt-mTOR axis by TGF-β1 and Col-1. These results also prompted us to determine whether mTOR was required for induction of stellate morphology by TGF-β1 and Col-1. To this end, A549 cells were cultured in rBM 3-D culture exposed to TGF-β1 and Col-1 in the presence or absence of Torin-1 (250 nM), an mTOR-specific inhibitor [25]. As expected, Torin-1 abrogated stellate morphology induced by TGF-β1 and Col-1 (Figure 4B). Moreover, Torin-1 attenuated the gene activation by TGF-β1 and Col-1 in that induction of the LOX and Myc genes was nearly abrogated, although induction of PAI-1 was refractory to Torin-1 (Figure 4C). These findings indicated that Src-mediated activation of the Akt-mTOR axis was required for stellate morphogenesis induced by TGF-β1 and Col-1.

## Discussion

The current study investigates the molecular mechanisms that mediate cancer progression promoted by the fibrotic tumor microenvironment using rBM 3-D culture of lung cancer cells. We aim to define the molecular mechanisms that mediate the tumor-promoting effects derived from the fibrotic tumor microenvironment.

rBM 3-D culture has been successfully applied to characterize molecular and cell biology of normal and transformed mammary epithelial cells for the past two decades [11-13]. In essence, rBM 3-D culture bears similar potential for investigation of lung biology because the lung and the breast share several key developmental and structural traits, such as branching morphogenesis during development and formation of alveoli [26]. Indeed, rBM 3-D culture of normal human lung alveolar type II epithelial cells promotes expression of the differentiation markers, such as surfactant protein C and formation of acini, an *in vitro* mimic of alveoli [16]. More importantly, over-expression of PPAR-γ, a tumor suppressor gene, can restore formation of acini in a poorly differentiated human lung cancer cell line in rBM 3-D culture [17]. Our findings strengthen the concept that rBM 3-D culture can be used to assess invasive and metastatic potential of lung cancer cells by comparing morphogenesis of four lung cancer cell lines with distinct tumorigenic behaviors *in vivo*. By and large, the well-differentiated lung adenocaricnoma cells, A549 and mK-ras-LE, form acini, whereas the more aggressive A549LC and LLC cells exhibit mass and stellate morphology (Figure 1). The diverse growth patterns of these four lung cancer cell lines in rBM 3-D culture are congruent to their disparate histology and tumorigenic potential *in vivo* (Figure 1). It is noteworthy that rBM 3-D culture reveals distinct morphogenesis between A549 (acinar morphology) and A549LC (mass morphology), whereas the two cell lines appear nearly identical in 2-D culture (data not shown). The morphological difference in rBM 3-D is also congruent to their distinct histology and tumorigenic activity *in vivo* (Figure 1, A & B). With further optimization and validation, rBM 3-D organotypic culture can be utilized as a surrogate to qualitatively and quantitatively assess tumorigenic properties of lung cancer cells.

One major advantage of rBM 3-D culture is that it allows assessment of tumor modulating cues derived from the tumor microenvironment [11-13]. As revealed in our study, TGF-β1 and Col-1 synergistically induce stellate morphology, a hallmark feature of invasive/metastatic cancer cells (Figures 1 &2) [15]. This combined exposure may recapitulate the fibrotic tumor microenvironment *in vivo* where lung cancer cells are simultaneously and constantly exposed to a variety of fibrogenic mediators [6-8]. Induction of stellate morphology by a combination of TGF-β1 and Col-1 is also consistent with a previous study in which provisional ECM, such as fibronectin and Col-1 potentiates epithelial-mesenchymal transition (EMT) of alveolar type II epithelial cells in response to TGF-β1 in 2-D culture [27]. Thus, stellate morphology induced by TGF-β1 and Col-1 can be perceived as a phenomenon of EMT in rBM 3-D culture, which allows investigation of EMT of lung cancer cells, a pivotal step towards invasion/metastasis in the context of ECM. In support of our notion, characterization of EMT using rBM 3-D culture has been proposed as a routine protocol based on initial success of this approach [28].

Our attempt to pinpoint the mediators of the synergistic induction of stellate morphology by TGF-β1 and Col-1 results in limited success. Nevertheless, we identify the signaling pathway and target genes activated by the TGF-β1 arm, which is not sufficient, but required for transition from acinar to stellate morphology (Figures 2, 3, 4). Specifically, the Src kinase activity is required for induction of stellate morphology and activation of gene expression by TGF-β1 in the presence or absence of Col-1 (Figures 2 &3). Similarly, the Src kinase activity appears to be essential for activation of the Akt-mTOR axis by TGF-β1 in the presence or absence of Col-1 (Figures 2 and 4). Besides the inducible stellate morphogenesis, the Src kinase activity appears to be required for native stellate morphogenesis of the invasive/metastatic cancer cell lines because inhibition of the Src kinase activity abrogates stellate morphogenesis of the invasive/metastatic LLC, 4T1, and MDA-MB231 cells (unpublished observations).

Despite similar distortion of acinar morphogenesis, only TGF-β1, but not Col-1 stimulates the expression of the MYC, LOX, and PAI-1 (Figures 1 &3). It is conceivable that Col-1 employs an alternative gene expression program to disrupt acinar morphogenesis. In support of this notion, Col-1 stimulates the expression of the oncogenic miR-21 gene in rBM 3-D culture, which is not observed in lung cancer cells exposed to TGF-β1 (unpublished observations) [29]. Among the TGF-β1-activated tumor promoting genes, LOX exhibit an Src- and mTOR-dependence and a strong correlation to stellate morphology (Figures 3 &4) [9]. These findings suggest a novel mechanism for the elevated expression of LOX in human lung cancer in that TGF-β1 induces the expression of LOX in lung cancer cells via the Src-Akt-mTOR axis. It is also conceivable that the TGF-β1-induced expression of LOX in rBM 3-D culture crosslinks the supplemented Col-1 to substantially increase the stiffness of rBM 3-D culture and thereby mediates synergistic induction of stellate morphology by TGF-β1 and Col-1. Among the three genes examined upon blockade of Src and mTOR, PAI-1 appears to be refractory to inhibition of mTOR, whereas inhibition of Src diminishes activation of all three genes (Figures 3 &4). This suggests that mTOR mediate only part of the gene activation program activated by Src upon exposure to TGF-β1. This observation could also be attributed to the SMAD3 binding motif in the PAI-1 promoter that induces the expression of PAI-1 through SMADs, the canonical TGF-β pathway and delivers resistance to blockade of the non-canonical TGF-β pathways, such as mTOR [30].

In summary, we demonstrate that the fibrogenic mediators derived from the tumor microenvironment promote stellate morphogenesis of lung cancer cells. Our results further suggest that the Src-Akt-mTOR axis, a group of promising therapeutic targets in lung cancer, acts as a signal transducer of the fibrotic tumor microenvironment [22,23,31]. Our work warrants further investigation to elucidate the molecular mechanisms that mediate synergistic induction of stellate morphology by TGF-β1 and Col-1. These findings also strongly suggest that rBM 3-D culture can serve as an ideal platform for swift and cost-effective screening of therapeutic candidates at the interface of the tumor and its microenvironment.

## Methods

### Reagents and plasmids

PP2, an Src specific inhibitor, was purchased from Calbiochem (San Diego, CA). Matrigel was purchased from BD Biosciences (Rockville, MD). Rat Col-1 was purchased from Sigma (St. Louis, MO). Recombinant human TGF-β1 was obtained from R&D Systems (Minneapolis, MN). A dominant-negative chicken Src-K295R mutant expressing retroviral vector (dnSrc) and its backbone (pLNCX) were kindly provided by Dr. Brugge at Harvard University [32,33]. Torin1, an mTOR-specific inhibitor was a gift from Dr. Sabatini at MIT [25]. Invitrogen (Carlsbad CA) provided the antibodies specific for total and phosphorylated (Tyr861) FAK. Cell Signaling (Danvers MA) provided the antibodies specific for total and phosphorylated Src (Tyr416), Akt (Ser473), mTOR (Ser2448), and p70 S6K (Thr389).

### Cell culture

A549 cells, a human lung adenocarcinoma cell line were obtained from ATCC (Manassas VA) and cultured as previously described [34,35]. A549LC cells were derived from parental A549 cells using a murine model of lung metastasis [36,37]. Briefly, A549 cells (10^6^ cells/mouse) were injected via the jugular vein into adult female beige-SCID mice (Charles River). Four months after injection, lungs were inspected and one metastatic nodule was excised, disaggregated and established in culture. The dnSrc expressing variant of A549LC (A549LCdnSrc) and its matching backbone vector variant (A549LCvec) were generated using retroviral transduction as we previously described [38]. mK-ras-LE cells, a murine lung epithelial cell line, were established from a tumor bearing lung of a *K-ras*^*LA1*^ transgenic mouse and cultured in RPMI-1640 as described elsewhere [20,29]. Lewis lung carcinoma cells (LLC), a metastatic murine lung cancer cell line, were purchased from ATCC (Manassas, VA) and cultured in DMEM.

### rBM 3-D organotypic culture and image analysis

rBM 3-D organotypic culture was employed because of the prior success of this approach in characterizing differentiation of both primary and transformed lung epithelial cells [11-13,16,17]. Briefly, the lung cancer cells were seeded in an overlay fashion on a layer of Matrigel on day zero. The culture medium containing 4% Matrigel (volume/volume) was replaced every other day. Formation of acini was monitored for twelve days prior to harvest for image, RNA, and protein analyses. The cultured cells were visualized using fluorescent staining for filamentous actin with Alexa 488 conjugated phalloidin (Invitrogen, Carlsbad CA). The images were captured using confocal fluorescent or phase contrast microscopy as we previously described [26,39]. In the selected cultures, various combinations of TGF-β1 (5 ng/ml), Col-1 (1.5 μg/ml), and Torin-1 (250 nM) were added to rBM 3-D culture.

### RNA extraction and quantitative RT-PCR

Total cell RNA was extracted from rBM 3-D culture using TRIzol per the provider’s instructions (Invitrogen, Carlbad CA). The expression of each gene of interest was determined using quantitative RT-PCR on an iCycler (BIO-RAD, Hercules CA) and compared across the groups as described else where [40]. The sequences of each pair of primers were listed in Additional file 1: Table S1.

### Immunoblots

Total cell protein was extracted using RIPA buffer supplemented with Protease and Protein Phosphatase Inhibitor Cocktails (Sigma, St. Louis MO) after A549 cells and their variants were extracted from rBM 3-D culture [26]. The expression of the total and phosphorylated proteins of interest was determined using immunoblots as described we previously described [34].

### Implantation of lung cancer cells

All mouse studies were carried out following the animal protocol approved by the Institute Animal Care and Use Committee at Tulane University School of Medicine. Subcutaneous implantation of human and mouse lung cancer cells (2 × 10^6^ cells/mouse) into male nude and syngeneic mice (Charles River, Wilmington, MA) was carried out as we previously described [36,41]. Each group of tumor graft consisted of 7 mice. Tumor growth was monitored daily after implantation. The tumor mass was dissected from mice at four weeks after implantation and processed for weighing and H&E staining.

### Statistical analysis

When presented, means and standard deviations were obtained from 3 independent experiments. A *P* value between any two selected groups was determined using unpaired two-tailed Student's T-test (GraphPad Prism, Version 5).

## Abbreviations

ECM: Extracellular matrix; LOX: Lysyl-oxidase; TGF-β1: Transforming growth factor-β1; Col-1: Type I collagen; mTOR: Mammalian target of rapamycin; PAI-1: Plasminogen activator inhibitor-1.

## Competing interests

The authors declare no competing interests.

## Authors’ contribution

HTN carried out rBM 3-D culture, image analysis, qRT-PCR, and immunoblots. YZ carried out qRT-PCR experiments and immunoblots. LS and YZ carried out xenograft studies of A549 and mK-ras-LE cells. SPK and JKK generated A549LC cells and carried out xenograft studies of A549LC cells. ZY carried out xenograft studies of LLC cells. BS conceived the study and wrote the manuscript. All authors read and approved the final manuscript.

## Supplementary Material

Additional file 1: Table S1 Primers. Figure S1 Tumor growth of subcutaneously implanted A549 and A549LC cells. Click here for file
