# Supplementary material for: Src-mediated morphology transition of lung cancer cells in three-dimensional organotypic culture
Source: Cancer Cell Int. 2013 Feb 14;13:16. doi: 10.1186/1475-2867-13-16 (PMC3626791; doi:10.1186/1475-2867-13-16)

Table 1. Primers:

Human LOX (GenBank ID: NM_002317)
Forward primer 5' — GATACGGCACTGGCTACTTCCA — 3'
Reverse primer 5' — GCCAGACAGTTTTCCTCCGCC — 3'

Human PAI-1 (GenBank ID: NM_000602)
Forward primer 5' — CTCATCAGCCACTGGAAAGGCA — 3'
Reverse primer 5' — GACTCGTGAAGTCAGCCTGAAAC — 3'

Human Myc (GenBank ID: NM_002467)
Forward primer 5' — CCTGGTGCTCCATGAGGAGAC — 3'
Forward primer 5' — CAGACTCTGACCTTTTGCCAGG — 3'

Figure 1. Tumor Growth of Subcutaneously Implanted A549 and A549LC cells
A549 and A549LC cells were grafted into the flank of nude mice as described in the section of Materials and Methods. The tumors were dissected and wet weight of each tumor was recorded. The weight of the tumors formed by A549 and A549LC cells was compared. The mean and standard deviations were obtained from 7 grafted mice of each A549 variant. A *P* value of 0.0678 was obtained using unpaired two-tailed student T-test (Prizm).

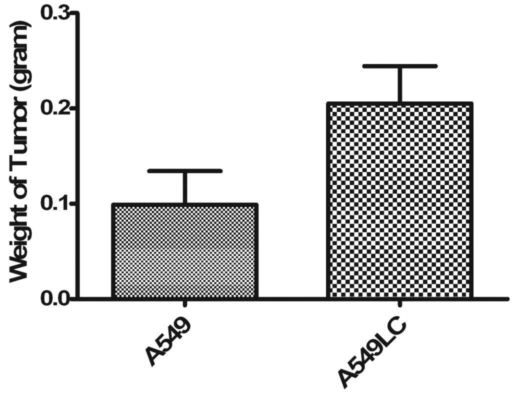

Supplement: Additional file 1: Table S1 — Primers. Figure S1 Tumor growth of subcutaneously implanted A549 and A549LC cells. [file 1475-2867-13-16-S1.pdf]
